# Papillary Muscle Lipoma in a Teenage Patient With Review of the Literature

**DOI:** 10.1016/j.case.2023.04.003

**Published:** 2023-05-23

**Authors:** Mohammad Sahebjam, Sahar Asl Fallah, Aryan Ayati, Mahkameh Farmanesh

**Affiliations:** a Cardiovascular Diseases Research Institute, Tehran University of Medical Sciences, Tehran, Iran; b Department of Echocardiography, Tehran Heart Center, Tehran University of Medical Sciences, Tehran, Iran

**Keywords:** Papillary muscle lipoma, Transthoracic echocardiography, Cardiovascular magnetic resonance imaging

## Abstract

• Cardiac lipoma is exceedingly rare in both adult and pediatric populations.
• Only a few cases of papillary muscle lipoma have been reported.
• TTE is the first step in detecting a cardiac mass.
• CMR contributes to the diagnosis of cardiac lipoma.
• There are no specific guidelines for the treatment of papillary muscle lipoma.

## Graphical abstract

**Figure:**
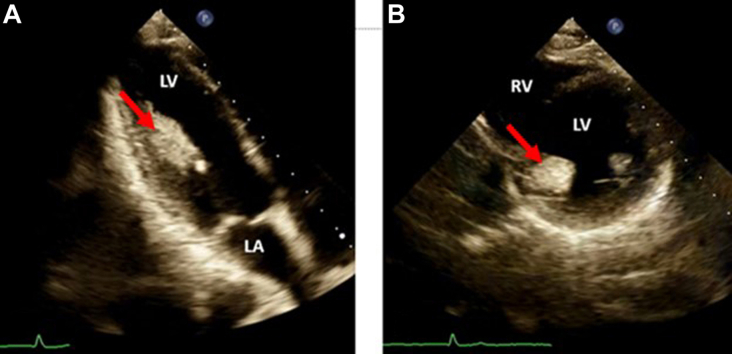

## Introduction

Cardiac lipoma, which is exceedingly rare in both adult^1^ and, particularly, pediatric^2^ populations, has a broad spectrum of presentations. However, most are asymptomatic and detected in an investigation for other causes.^3^ Due to the extremely low prevalence of cardiac lipomas arising from unusual papillary muscle sites,^4^, ^5^, ^6^, ^7^ the treatment is challenging, especially in the young.

## Case Presentation

**Figure 1:**
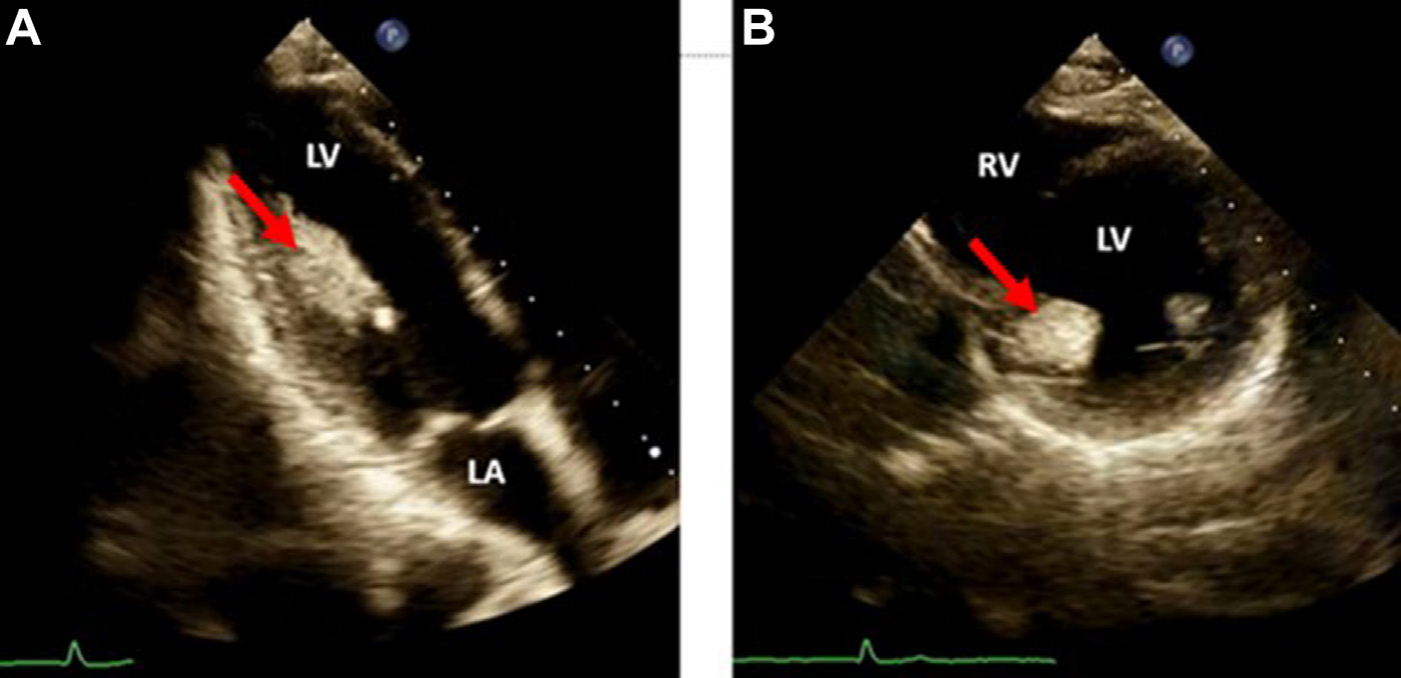
Two-dimensional TTE, rotated parasternal long-axis view, systolic phase **(A)** and parasternal short-axis diastolic phase **(B),** demonstrates a homogeneously hyperechoic mass *(arrows)* without echolucency involving the posteromedial papillary muscle; the anterolateral papillary muscle appears normal. *LA*, Left atrium; *LV*, left ventricle; *RV*, right ventricle.

**Figure 2:**
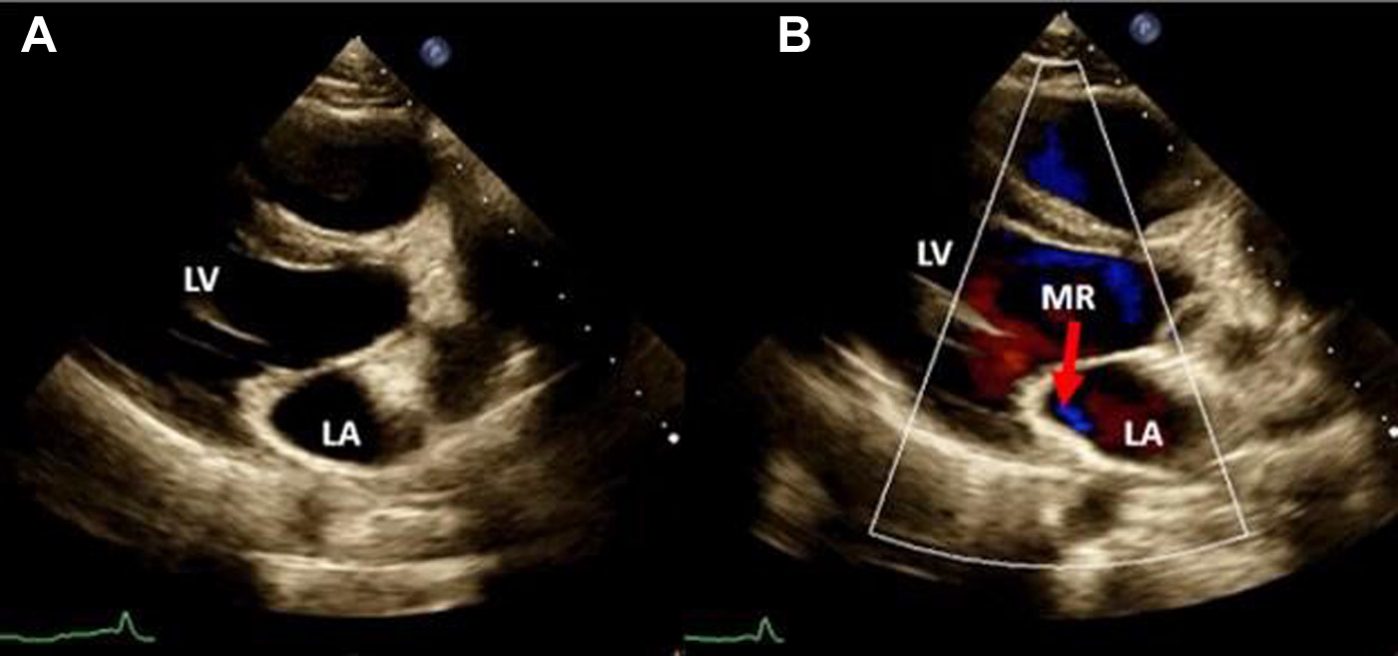
Two-dimensional TTE parasternal long-axis view, systolic phase without **(A)** and with **(B)** color flow Doppler, demonstrates mild eccentric, laterally directed, mitral regurgitation (MR; *arrow*). *LA*, Left atrium; *LV*, left ventricle.

**Figure 3:**
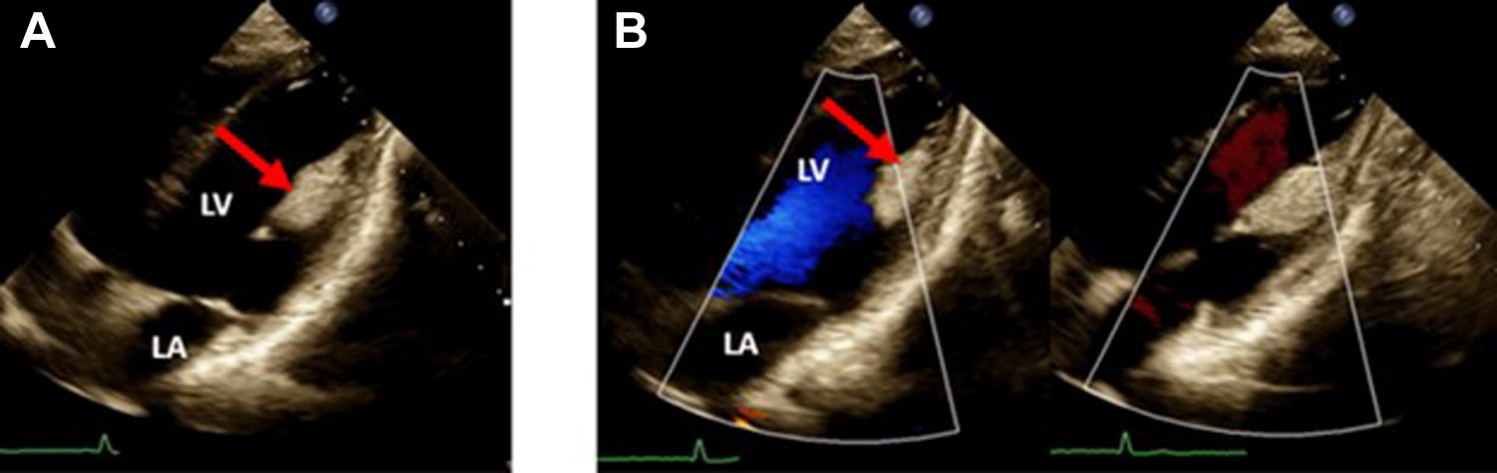
Two-dimensional TTE, rotated apical 4-chamber view, systolic phase without **(A)** and both systolic/diastolic phases with **(B)** color flow Doppler, demonstrates the echo-bright, posteromedial papillary muscle lipoma *(arrows)* without blood flow signal within the mass. *LA*, Left atrium; *LV*, left ventricle.

**Figure 4:**
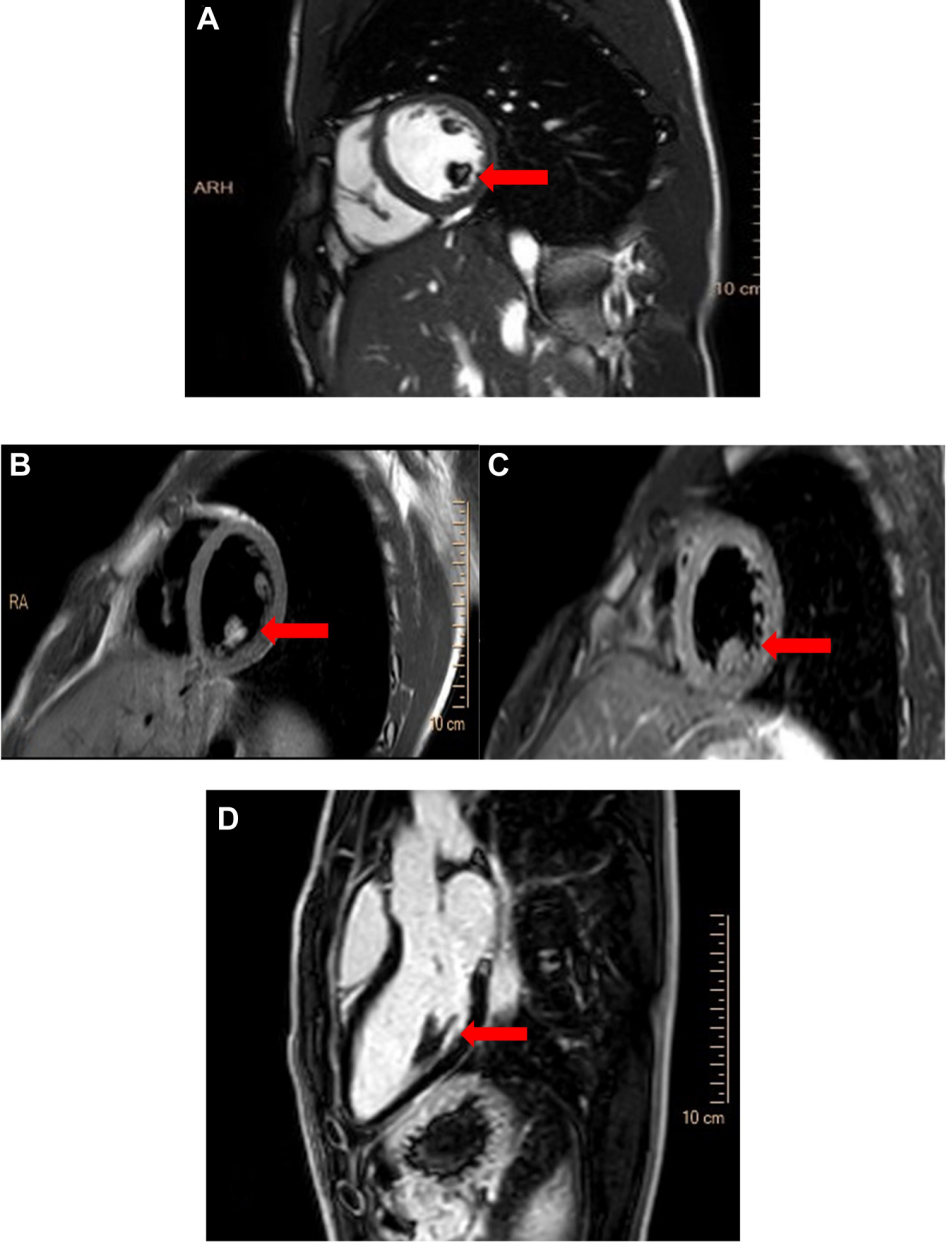
CMR imaging of the posteromedial papillary muscle lipoma *(arrows)* with bSSFP sequence **(A)**, short tau inversion recovery sequence **(B)**, fat saturation sequence **(C),** and late gadolinium enhancement sequence **(D)**. *LA*, Left atrium; *LV*, left ventricle; *RA*, right atrium; *RV*, right ventricle.

A 14-year-old boy with a chief complaint of atypical (chest wall) chest pain was referred to our department for transthoracic echocardiography (TTE) and further evaluation. The patient denied any cardiac problems in their personal or familial medical history. The patient’s vital signs and physical examination were unremarkable. A TTE revealed a large homogeneously hyperechoic immobile mass without echolucency of the posteromedial papillary muscle and with normal appearance of the anterolateral papillary muscle (Figure 1, Videos 1 and 2). The left ventricle was normal in size and function, with mild eccentric to lateral wall mitral regurgitation (Figure 2, Video 3). Color flow Doppler imaging showed no flow within the mass (Figure 3). In the cardiovascular magnetic resonance imaging (CMR) study (Figure 4, Videos 4 and 5), the posteromedial papillary muscle showed India ink artifact (type 2 chemical shift artifact) in balanced steady-state free precession (bSSFP) sequences, high signal intensity in T2-based sequences, and isointense signal intensity in short tau inversion recovery sequence as a T2 fat suppression sequence, all of which indicate fat-containing tissue. In the gadolinium study, no early gadolinium enhancement in the first pass perfusion (Video 6) or late gadolinium enhancement was detected, which also favors lipoma in the posteromedial papillary muscle.

## Discussion

Primary cardiac tumors are rare benign tumors. In adults, the incidence is between 0.2% and 0.4%, among which lipoma is an extremely rare benign tumor that accounts for 8% in autopsy series.^1^ In patients younger than 18, rhabdomyoma is the most common cardiac tumor, followed by fibroma. The primary malignant tumors include rhabdomyosarcoma and fibrosarcoma.^2^ Lipoma may occur in individuals of different ages and gender; however, it is more common among elderly patients.^1^^,^^8^ Moreover, lipoma constitutes a minority of primary cardiac tumors in children, and only a few pediatric cases diagnosed with cardiac lipoma have been reported.^2^ Lipomas are often intracardiac sessile masses originating primarily from the subendocardium (50%), followed by an equal distribution of the subepicardium and myocardium (25%).^9^ In a systematic review in 2021, lipomas were mostly found to be located in the right atrium (42.4%) and left ventricle (33.3%) in different sizes.^10^ We found only 3 cases of cardiac lipoma arising from papillary muscle through a PubMed search reviewed in Table 1.

**Table 1:** A summary of previous reports of a papillary muscle lipoma

Author	Koshy *et al.*^5^	Prestipino *et al.*^6^	Kim *et al.*^7^
Publish date	2010	2016	2018
Gender	Female	Female	Male
Age	67	42	42
Location	Anterolateral papillary muscle	Anterolateral papillary muscle	Posteromedial papillary muscle
Symptoms	Numbness on the right side with blurry vision	Asymptomatic	Asymptomatic
History of illness	Temporal malignant melanoma	None	None
Treatment	Surgical resection	Close follow-up	Surgical resection and mitral valve replacement

In the review of these 3 cases, 2 had lipoma in the anterolateral papillary muscle and 1 in the posteromedial papillary muscle, as in the mentioned case. Koshy *et al.*^5^ reported the first case in 2010, a middle-aged woman with a history of melanoma who underwent anterolateral papillary muscle lipoma resection due to an ischemic event. In 2016, Prestipino *et al.*^6^ reported a middle-aged woman who was incidentally diagnosed with anterolateral papillary muscle lipoma during a checkup assessment. The patient was closely monitored due to the absence of symptoms. Kim *et al.*^7^ published the latest case of cardiac lipoma in 2018 and reported a middle-aged man with posteromedial papillary muscle lipoma who underwent surgery for a cardiac lipoma tumor as well as mitral valve replacement. Although a cardiac lipoma is usually asymptomatic, it may be incidentally detected while investigating other disorders.^4^ However, depending on its location and size, it can cause symptoms such as chest pain, palpitations, arrhythmia, or even sudden cardiac death due to coronary artery or valve obstruction.^11^ Transthoracic echocardiography is the first step in detecting a cardiac mass. If a cardiac mass cannot be defined or have a clear characterization through TTE, it is recommended that other imaging methods be utilized. Cardiac computed tomography and CMR have complementary values for evaluating cardiac masses. On cardiac computed tomography with and without contrast, a Hounsfield unit of less than –50 and enhancement after contrast administration suggest benign lipoma. Positron emission tomography can provide precise diagnostic information in determining cardiac tumors.^3^^,^^10^ The echocardiographic findings of different cardiac masses are summarized in Table 2.^10^, ^11^, ^12^, ^13^, ^14^, ^15^

**Table 2:** Typical echocardiographic findings of cardiac masses

Rhabdomyoma	Homogenous echogenicity; hyperechogenic, hyperlucent central areas due to foci of necrosis or calcification; usually multiple, small in size; located in ventricles; can mimic diffuse myocardial thickening
Myxoma	Hyperechoic; smooth or villi surface, including lucencies; mobile mass attached to the endocardial surface by a stalk; located in the left atrium, atrial septum
Endocardial fibroelastosis	High echo brightness of the endocardium; thickening of the endocardium due to the proliferation of fibrous and elastic tissue
Lipoma	Homogeneous due to fat content; hyperechoic within cardiac chambers (if located in pericardial space = hypoechoic); broad base, usually immobile; located on all sites
Fibroma	Hyperechoic; homogenous; central calcification; located in the ventricles, ventricular septum
Rhabdomyosarcoma	Homogenous echogenicity; hyperechogenic; hyperlucent central areas due to foci of necrosis or calcification; located in ventricles

*LA*, left atrium.

Acoustic characteristics alone cannot routinely differentiate benign lipomas from other malignant tumors, and CMR is the preferred complementary noninvasive imaging technique to assist in diagnosing cardiac masses since it offers improved tissue differentiation. Due to the very low prevalence of cardiac lipoma, there is no specific guideline for the treatment of such tumors. However, in symptomatic patients, surgery will be indicated.^4^ Close follow-up by imaging modalities can detect any recurrence or changes in tumor size.^10^

This case study presents a young patient with an unusual location of a cardiac lipoma in the posteromedial papillary muscle. After extensive consultation with the heart team, considering the mass size and the absence of clinical or hemodynamic symptoms, it was concluded that we should proceed with conservative management consisting of serial clinic visits, physical examinations, and TTE. Should there be any changes in the TTE findings, a CMR should be performed to help guide treatment decisions.

## Conclusion

Transthoracic echocardiography can primarily detect papillary muscle lipoma, and CMR can provide more details as a complementary modality. Careful considerations are required according to mass location, size, and clinical symptoms to select an appropriate treatment method.

## Consent Statement

Complete written informed consent was obtained from the patient (or appropriate parent, guardian, or power of attorney) for the publication of this study and accompanying images.

## Ethics Statement

The authors declare that the work described has been carried out in accordance with The Code of Ethics of the World Medical Association (Declaration of Helsinki) for experiments involving humans.

## Funding Statement

The authors declare that this report did not receive any specific grant from funding agencies in the public, commercial, or not-for-profit sectors.

## Disclosure Statement

The authors report no conflict of interest.

## Supplementary Data

Video 1 Two-dimensional TTE, rotated parasternal long-axis view, demonstrates a large homogeneously hyperechoic immobile mass of the posteromedial papillary muscle. Normal left ventricle systolic function is also noted.

Video 2 Two-dimensional TTE, parasternal short-axis at the level of papillary muscle view, demonstrates an echo-dense mass-like lesion arising from posteromedial papillary muscle with normal appearance of the anterolateral papillary muscle.

Video 3 Two-dimensional TTE, parasternal long-axis view with color flow Doppler, demonstrates mild eccentric, posteriorly directed mitral regurgitation (MR).

Video 4 Two-chamber cine bSSFP CMR sequence demonstrates normal left ventricle size and function with a focally prominent posteromedial papillary muscle including the lipoma; an India ink (type 2 chemical shift) artifact is seen at the border of this mass indicating fat-containing tissue.

Video 5 A stack of 3-chamber cines, bSSFP CMR sequences demonstrates the focally prominent posteromedial papillary muscle lipoma with the India ink (type 2 chemical shift) border artifact indicating fat-containing tissue.

Video 6 Mid left ventricular, CMR first-pass perfusion sequence demonstrates normal myocardial perfusion and a focally dark mass that represents nonperfusion of the posteromedial papillary muscle with the lipoma.
